# Effects of Dissolving Solutions on the Accuracy of an Electronic Apex Locator-Integrated Endodontic Handpiece

**DOI:** 10.1155/2013/475178

**Published:** 2013-11-26

**Authors:** Yakup Ustun, Ozgur Uzun, Ozgur Er, Murat Maden, Fatma Yalpı, Burhan Can Canakci

**Affiliations:** ^1^Department of Endodontics, Faculty of Dentistry, Erciyes University, 38039 Kayseri, Turkey; ^2^Department of Endodontics, Faculty of Dentistry, Gazi University, 8th Street Emek-Ankara, 06510 Ankara, Turkey; ^3^Department of Endodontics, Faculty of Dentistry, Suleyman Demirel University, 32060 Isparta, Turkey

## Abstract

The effects of three dissolving agents on the accuracy of an electronic apex locator- (EAL-) integrated endodontic handpiece during retreatment procedures were evaluated. The true lengths (TLs) of 56 extracted incisor teeth were determined visually. Twenty teeth were filled with gutta-percha and a resin-based sealer (group A), 20 with gutta-percha and a zinc oxide/eugenol-based sealer (group B), and 16 roots were used as the control group (group C). All roots were prepared to TL. Guttasolv, Resosolv, and Endosolv E were used as the dissolving solutions. Two evaluations of the handpiece were performed: the apical accuracy during the auto reverse function (ARL) and the apex locator function (EL) alone. The ARL function of the handpiece gave acceptable results. There were significant differences between the EL mode measurements and the TL (*P* < 0.05). In these comparisons, Tri Auto ZX EL mode measurements were significantly shorter than those of the TL.

## 1. Introduction

Root canal-treated teeth may require orthograde revision in the case of a persistent infection or following reinfection of the root canal [1]. Although endodontic surgery offers more favourable initial success, nonsurgical retreatment yields a more favourable long-term outcome in these failed cases [1]. A retreatment procedure in endodontic practice requires complete removal of the original root filling materials and enlarging and repreparing the root canal prior to refilling [1, 2]. Many techniques have been suggested for this purpose, including the use of rotary NiTi instruments [3, 4] and root canal filling-dissolving solvents [2, 5].

Furthermore, the use of rotary instruments with dissolving solvents may facilitate the removal of root canal filling materials and the repreparation of the root canals in clinical practice. Three such solvents are Endosolv E (Septodont, Saint-Maur-des-Fossé, France), Resosolv (Pierre Rolland, Merignac, France), and Guttasolv (Septodont), which contain primarily tetrachloroethylene, dimethylformamide, and eucalyptol, respectively. The manufacturers recommend the use of Endosolv E when zinc oxide/eugenol-based sealers have been used as the root canal sealer, Resosolv when resin-based sealers were used, and Guttasolv when gutta-percha was used as the root canal filling material. 

Accurate detection of the root canal terminus and the precise calculation of working length (WL) are critical in retreatment procedures to reduce the probability of insufficient removal of root filling material or of damaging the periapical tissues by instrumentation beyond the tooth [6, 7]. Additionally, the continuous monitoring of WL is important during removal of filling materials and repreparation as the WL may vary during the procedure, especially in curved canals [7]. Moreover, when using radiographic methods to obtain the WL in retreatment cases, the actual position of the file tip can be masked by the remnants of the filling materials and it may be necessary to eliminate more material from the apical zone to see the tip of the file clearly For these reasons, combinations of an electronic apex locator (EAL) and endodontic handpieces can be used in orthograde root canal retreatment cases to remove the filling materials and to reprepare the root canal. 

The Tri Auto ZX (Morita Corp., Kyoto, Japan) is one such combined device; the Root ZX (Morita Corp.), one of the most popular EALs, is installed in a torque-controlled and cordless handpiece. In this system, the instrument electrode is installed in the head of the handpiece and is connected to the EAL through the handpiece. The rotation speed of the instrument can be adjusted between 150 and 300 rpm. The Tri Auto ZX has three automatic mechanisms: auto-start-stop, auto-torque-reverse, and autoapical reverse. Previous studies have evaluated some features of this combined device under different *in vitro* and *in vivo* conditions [7–10]. Some of these studies have focused on the use of Tri Auto ZX in retreatment procedures [7, 9]; however, there is to date no published evaluation of the possible effects of commonly used dissolving solvents (Guttasolv, Endosolv E, and Resosolv) on the accuracy of the Tri Auto ZX in retreatment procedures.

Thus, the aim of this *in vitro* study was to evaluate the effects of three dissolving agents on the accuracy of an EAL and endodontic handpiece combined device during an orthograde retreatment procedure.

## 2. Materials and Methods

### 2.1. Sample Preparation

In total, 56 freshly extracted human maxillary incisor teeth, stored in saline, were used. The teeth were evaluated by obtaining mesiodistal and buccolingual radiographs and an operating microscope at ×10 magnification (Opmi Pico, Carl Zeiss, Germany) to determine that they had noncomplicated root canal anatomy, mature root formation, and no external root resorption, cracks, or fractures along the roots. All crowns were cut at the cementoenamel junction with a diamond disc to simplify access to the root canal and to provide a stable reference for all measurements. Root canal patency was controlled with a size 10 K-file (Mani Inc., Tochigi-Ken, Japan) in each root.

### 2.2. Assessment of the True Length

The true length (TL) was measured visually with the help of an operating microscope at ×10 magnification, as described by Thomas et al. [11]. A size 10 stainless steel file (VDW, Antaeos, Munich, Germany) was placed into the root canal until the tip of the file reached the plane of the major foramen. The distance between the file tip and the stopper was measured with digital callipers (±0.01 mm accuracy). The measurements were repeated three times, and the average was taken as the raw length (RL). Then, 0.5 mm was subtracted from the RL measurements, and the calculated value was recorded as the TL.

### 2.3. Preparation of Root Canals

Each root was prepared using the ProTaper System (Dentsply, Maillefer, Ballaigues, Switzerland) to TL. S1, S2, F1, and F2 files were used, according to the manufacturer's recommendations, with an endodontic motor (X-Smart, Dentsply). The master apical file (MAF) was F2 for all roots. The root canals were irrigated with 2.5% NaOCl after each change of instrument. For the final irrigation, 3 mL of 17% EDTA was used for 1 min, followed by 3 mL of 2.5% NaOCl and 3 mL of distilled water. The root canals were then dried with paper points. 

The roots were randomly divided into two experimental groups (*n* = 20 each) and a control group (*n* = 16; Table 1).

### 2.4. Penetration of Root Fillings and Assessment of Electronic Working Lengths (ARL and EL)

In the present study, two evaluations were performed for the Tri Auto ZX device. The first was the apical accuracy during the autoreverse function (“ARL”), and the second was only the apical accuracy during the EAL function (“EL”).

#### 2.4.1. Group A

Twenty roots were filled with lateral compaction using a size F2 master gutta-percha cone (Dentsply) and an epoxy resin-based (AH Plus, Dentsply DeTrey, Konstanz, Germany) root canal sealer. Lateral compaction was achieved in each canal using accessory gutta-percha cones (Diadent Group International, Chongchong Buk Do, Korea) and a finger spreader. Group A was randomly divided into two subgroups (*n* = 10 each). The roots were stored at 100% humidity at 37°C for 1 week to set the sealers.

*(1) Subgroup A1 (Guttasolv Group)*. The roots were embedded in an alginate testing model [12]. The coronal part of the root canal fillings (approximately 3 mm) was removed using a Gates Glidden drill (Mani Inc., Japan) to create a reservoir for the solvent. In this group, Guttasolv was used (0.2 mL injected into the root canal and a 1 min wait at the beginning). The “automatic apical reverse function” (ARL) of the device was set to start at the 0.5 setting. To compare the accuracy of the device, the length of the instrument at which the ARL function was initiated during active (rotary) penetration was measured. Then, a second electronic measurement of canal length (EL) was obtained when the instrument was reinserted into the canal passively (without rotation). Mean differences between each electronic measurement and TL were compared. The Tri Auto ZX was used according to the manufacturer's recommendations. A ProTaper F3 instrument attached handpiece was adjusted to the high torque level and inserted into the root canal and the Tri Auto ZX device was operated. The rotating instrument was advanced down the canal to penetrate the softened gutta-percha and sealer without exerting excessive force. After three or four pecking motions, the file was removed from the canal and cleaned. At the same time, 0.2 mL solvent was injected into the canal again and left for 1 min. Then, the cleaned file was inserted into the root canal and the Tri Auto ZX device was operated again. When a beeping sound was heard, the integrated root canal length measurement device of the Tri Auto ZX determined that the instrument tip was at the 0.5 level. At this length and just before the instrument began to rotate in the opposite direction, the instrument was stopped by the operator. Then, the rubber stop on the instrument was adjusted to the flat coronal surface. The rubber stop was fixed to the instrument with a flowable light-curing resin (GrandioFlow, Voco GmbH, Germany). The instrument was removed and the distance between the rubber stop and the file tip was measured using the digital callipers (±0.01 mm accuracy); this length was referred to as A1_ARL_. Then, another F3 instrument was attached to the device and inserted into the canal passively, without rotary motion, until the integrated Tri Auto ZX device determined that the tip was again at the 0.5 level. The rubber stop of the instrument was fixed with GrandioFlow and the length measured using the digital callipers (±0.01 mm accuracy); this length was referred to as A1_EL_. In total, 0.4 mL Guttasolv solvent was used in this subgroup.

*(2) Subgroup A2 (Resosolv Group)*. The roots in this group were reprepared using the same method as for subgroup A1. The MAF was F3 for the retreatment procedure. In total, 0.4 mL of Resosolv solvent was used, with the same method as for subgroup A1, during the procedure. The whole operation and the ARL and EL measurement procedure was as those used for subgroup A1. The electronic lengths are referred to as A2_ARL_ and A2_EL_ in this subgroup.

#### 2.4.2. Group B

Twenty roots were filled with lateral compaction using a size F2 master gutta-percha cone and a zinc oxide/eugenol-based (Tubliseal, Kerr, Scafati, Italy) root canal sealer. Lateral compaction was achieved in each canal using accessory gutta-percha cones and a finger spreader. Group B was randomly divided into two subgroups (*n* = 10 each). The roots were stored at 100% humidity at 37°C for 1 week to set the sealers.

*(1) Subgroup B1*. The samples were reprepared using the same method as for subgroup A1. The MAF was the same (F3) as for subgroup A1. In total, 0.4 mL of Guttasolv solvent was used. The procedure used for subgroup A1 was repeated for ARL and EL measurements in subgroup B1. The electronic lengths are referred to as B1_ARL_ and B1_EL_ in this subgroup_._

*(2) Subgroup B2*. The samples were reprepared using the same method as for subgroup A1. The MAF was F3 for the retreatment procedure. In total, 0.4 mL of Endosolv E solvent was used. The procedure used for subgroup A1 was repeated for ARL and EL measurements in subgroup B2. The electronic lengths are referred to as B2_ARL_ and B2_EL_ in this subgroup_._

#### 2.4.3. Group C (Controls): Negative Control (Control Group C1)

Four roots were prepared with the ProTaper system and the MAF was F2. These four samples were left unfilled and no solvent was applied to the root canals. A F3 file was attached to the Tri Auto ZX and samples were reprepared using the same method as for subgroup A1. The procedure used for subgroup A1 was repeated for ARL and EL measurements in control group C1. The electronic lengths are referred to as C1_ARL_ and C1_EL_ in this control group. 

*Positive Control*. Twelve roots were prepared with the ProTaper system and the MAF was F2. They were then left unfilled. Endosolv E (0.4 mL; *control group C2*), Resosolv (0.4 mL; *control group C3*), and Guttasolv (0.4 mL; *control group C4*) solvents were injected into four roots per group. A F3 file was attached to the Tri Auto ZX and samples were reprepared using the same method as that used for subgroup A1. The procedure used for subgroup C1 was repeated for ARL and EL measurements in control groups C2, C3, and C4.

All measurements in all groups were performed by an experienced operator who was blinded to the TL measurements. Measurements were repeated three times for each tooth to ensure reproducibility, and the mean of the three measurements was used.

### 2.5. Statistical Analyses

All statistical analyses were performed using the SPSS software (ver. 13.0 for Windows; SPSS Inc., Chicago, IL, USA). The Shapiro-Wilks normality test and Levene's variance homogeneity test were applied to the data. The data were found to be normally distributed, and there was homogeneity of variance among the groups. A paired *t*-test was used for statistical analyses. The Tri Auto ZX device's ARL and EL function measurements were compared with TL measurements of each root and with each other in all subgroups and control groups. The percentage of acceptable measurements recorded with Tri Auto ZX at a ±0.5 and ±1 mm tolerance margin was analysed by *χ* ^2^ test at the 0.05 significance level.

## 3. Results

There was no significant difference in comparisons between the Tri Auto ZX ARL measurements and the TL among the experimental groups (*P* > 0.05, Table 2). The Tri Auto ZX ARL measurements showed similar values to those obtained from the TL during the retreatment procedures with different root canal sealers and different solvents (Table 2).

In comparisons between the Tri Auto ZX EL measurements and the TL, there were significant differences in subgroup A2 (Resosolv group; *P* < 0.05), control C3 (Resosolv group; *P* < 0.05), and control C2 (Endosolv group; *P* < 0.05; Table 2). In these comparisons, Tri Auto ZX EL measurements were significantly shorter than the TL.

**Figure 1:**
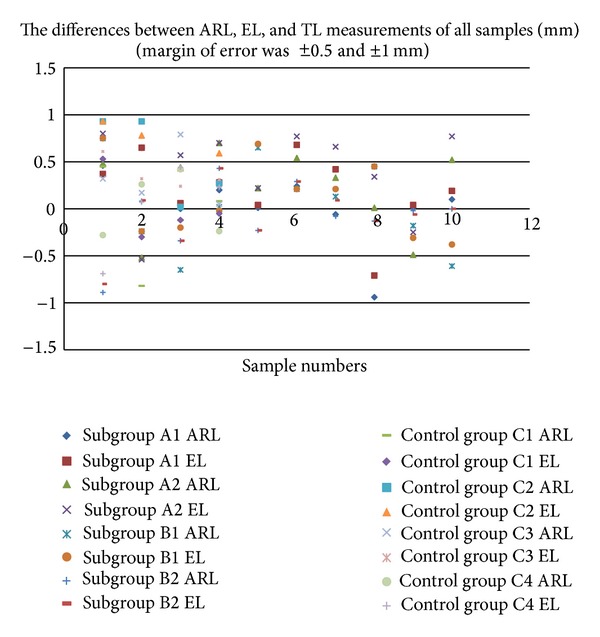
The differences between ARL, EL, and TL measurements of all samples (mm).

When the accuracy of the Tri Auto ZX was analysed at a ±0.5 mm margin of error, the accuracies of the Resosolv group (subgroup A2, 50%) and Endosolv control group (subgroup C2, 50%) were lower than those of the other subgroups in ARL measurements and the Resosolv groups (subgroup A2, 30%) and Endosolv control group (subgroup C2, 25%) were lower than those of the other subgroups in EL measurements (Table 1, Figure 1). At ±1 mm margin error, both ARL and EL functions had excellent accuracies (Table 1 and Figure 1).

## 4. Discussion

Working length (WL), defined as “the distance between a reference point from the coronal portion to the point at which canal instrumentation and filling should terminate” [13], is a critical factor for endodontic treatment and for retreatment outcomes [14]. Optimal healing occurs in infected roots when instrumentation and hermetic sealing are confined inside the root canal system [15]. Histological studies have shown that the presence of root canal filling materials in the periapical tissues may result in a persistent inflammatory condition [16]. Radiographic determination of the WL has limitations, such as distortion, shortening or elongation, and lack of a three-dimensional representation. In the search for more accurate WL measurements, methods of locating the apical foramen electronically have been developed. Current EALs have high reliability, high accuracy, and high reproducibility for WL determination, regardless of the electrolyte [17].

The Tri Auto ZX, an apex locator and endodontic motor combined handpiece, has five LED indicators on its control panel (APEX, 0.5, 1, 1.5, and 2). These indicators are used to set the level at which the autoreverse motion should begin in ARL mode and also to detect the position of the root canal instrument in EAL mode. Although in some previous studies, it was reported that EALs can determine a position within 0.5 mm of the major foramen more than 90% of the time [18, 19], there is little information regarding what position this “0.5” mark actually indicates [20]. Therefore, choosing the level for determining the WL seems mostly to depend on the personal experience and preferences of the practitioner, although Gulabivala et al. [21] and Nekoofar et al. [6] suggested that EALs should be used to achieve an “APEX” level reading for the greatest accuracy because the impedance characteristics for the canal, coronal to the apical foramen, cannot be calibrated accurately. However, the manufacturer claims that Tri Auto ZX does not require this calibration because a microprocessor corrects the calculated quotient. Moreover when using the Tri Auto ZX in clinical conditions, operators may not detect the “APEX” level first and then calculate the working length, as suggested, because the APEX level is the beginning of the periodontal ligament (PDL) in clinical conditions, when the device was active and the file attached was rotating in ARL mode. When the instrument tip reaches the APEX level (actually, the beginning of the PDL), it may damage the major foramen and PDL tissues, which we try hard to preserve for optimal healing conditions. So, one of the most important disadvantages of these combined devices is that WL cannot be calculated as accurately as a single EAL. Thus, in the present study, we subtracted 0.5 mm from the raw length of each tooth to determine the true length, and to simulate clinical conditions we set the ARL mode to start at the 0.5 level setting, and to make a scientific comparison we chose the 0.5 level in EAL mode.

In orthograde retreatment cases, use of a solvent is recommended to facilitate the removal of gutta-percha, by softening [4]. Chloroform is the most commonly used solvent because of its effectiveness [22]. Nevertheless, there are some limitations to its use. In particular, it has been suggested to be a potential carcinogen in uncontrolled use [22, 23] and it leaves a fine layer or film of softened gutta-percha [24]. In the present study, we did not use chloroform because of these reported disadvantages, but we used Guttasolv in two subgroups (A1 and B1) because gutta-percha was used for the root canal filling core material in all groups and Guttasolv is intended to soften gutta-percha material. We also used Resosolv, which was designed to be a resin-based sealer solvent, in the group in which a resin-based sealer was used (subgroup A2). Endosolv E, which was designed to be a zinc oxide/eugenol-based sealer solvent, was used in the group in which a zinc oxide/eugenol-based sealer was applied (subgroup B2).

A number of factors may influence the accuracy of EALs, such as the size of the apical foramen [25], the type and size of the measuring file [26], the irrigation solution used, and the electroconductivity of the pulp [27]. In the present study, two root canal sealers and three solvents were used, and we found no significant difference between the Tri Auto ZX ARL measurements and the TL in the presence of the different solvents in the root canal. There were significant differences between the Tri Auto ZX EL mode and the TL in the presence of solvents. Although it has been reported that EALs are reliable in the presence of different irrigants [28], the effects of root canal sealer solvents on EAL accuracy have not been evaluated before. Thus, we cannot compare the results of the present study with those studies. However, in one similar study, Uzun et al. [7] compared two different combined devices (TCM Endo V and Tri Auto ZX) for removing gutta-percha and sealer from filled root canals with chloroform solvent and reported that the ARL function of both devices must be used with caution when removing gutta-percha root fillings.

The size of the apical foramen [29] and the instrument used [30] have been reported to be important parameters for EAL accuracy. In the present study, we did not attempt to standardise the apical size of the samples, because it was not possible to find apically uniform-sized extracted teeth. Uniform-sized apical foramens can be generated by widening the roots progressively using bigger instruments, but with this technique, the apical anatomy is changed and the apically enlarged samples may not mimic real clinical conditions. In the present study, we chose similar-sized teeth and used the same size instruments for all samples to increases the reliability of our results.

Previous studies have reported that the orthograde retreatment technique should include additional enlargement of root canals beyond the initial canal preparation [2]. Thus, during retreatment procedures the MAF must be one or more sizes larger than the MAF used in the first preparations, as suggested by Taşdemir et al. [4]. In the present study, we used an F2 file for first preparation and an F3 file of the ProTaper system for repreparation of the root canals. 

The differences in ARL and EL measurements in the present study in the Resosolv and Endosolv groups may be attributable to the 0.5 level choices for the apical limit because previous studies reported that EALs are more accurate when the APEX level is chosen as the apical limit [6, 21]. Also, the electrical conductivity of the dissolving agents may be responsible for the shorter lengths and poorer accuracy in the Resosolv and Endosolv E groups. However, we have no reasonable explanation of the differing accuracies between the same solvent sample and control groups (between Resosolv subgroups A2 and C3 and between Endosolv E subgroups B2 and C2). Further study is needed to evaluate the effects of solutions' electrical conductivity on the accuracy of EAL and endodontic motor combined handpieces and to evaluate their effects *in vivo*.

## 5. Conclusions

Within the limitations of the present study, the ARL function alone of the Tri Auto ZX gave acceptable results in all groups and the EAL function gave acceptable results in some groups when the device was set at 0.5 as the autoreverse apical limit. Under clinical conditions, EAL—endodontic motor combined devices generally use ARL functions for continuous monitoring of WL, suggesting that clinicians can use the ARL function of the Tri Auto ZX device safely with Guttasolv, Endosolv E, and Resosolv solutions in retreatment cases.

## Figures and Tables

**Table 1:** The distribution of all experimental groups according to the materials used and the percentages of accuracy at ±0.5 and ±1.0 mm of all groups for ARL and EL modes according to true length (TL).

	Test samples				Test samples	Control groups				Control samples	Total
	Subgroup A1	Subgroup A2	Subgroup B1	Subgroup B2		C1	C2	C3	C4		
Filling material	Gutta + AH P	Gutta + AH P	Gutta + TubliSeal	Gutta + TubliSeal		Empty	Empty	Empty	Empty		
Dissolving Solution	Guttasolv	Resosolv	Guttasolv	Endosolv E		Free	Endosolv E	Resosolv	Guttasolv		
Sample number	10	10	10	10	40	4	4	4	4	16	56
Accuracy at ±0.5 mm (ARL)	80%	50%	60%	90%	72.5%	75%	50%	75%	100%	75%	73.75%
Accuracy at ±0.5 mm (EL)	70%	30%	80%	90%	67.5%	75%	25%	75%	75%	62.5%	65%
Accuracy at ±1 mm (ARL)	100%	100%	100%	100%	100%	100%	100%	100%	100%	100%	100%
Accuracy at ±1 mm (EL)	100%	100%	100%	100%	100%	100%	100%	100%	100%	100%	100%

**Table 2:** Sg: subgroup, M: mean, SD: standard deviation; subgroups' evaluations were done according to three different parameters (Tri Auto ZX ARL, TriAuto ZX EL, and TL). Different superscript letters show significant differences (*P* < 0.05).

	Group A		Group B		(+) Control			(−) Control
	Sg A1	Sg A2	Sg B1	Sg B2	C2	C3	C4	C1
	M ± SD	M ± SD	M ± SD	M ± SD	M ± SD	M ± SD	M ± SD	M ± SD
Tri Auto ZX ARL	12.35 ± 1.65	11.91 ± 0.63^B^	13.0 ± 1.59	12.68 ± 1.76	11.77 ± 0.69^D^	11.54 ± 0.29	10.80 ± 0.98^F^	12.2 ± 1.07
Tri Auto ZX EL	12.20 ± 1.71	11.73 ± 0.67^A^	12.94 ± 1.58	12.65 ± 1.78	11.62 ± 0.87^C^	11.52 ± 0.40	10.76 ± 1.12^E^	12.14 ± 0.99
TL	12.42 ± 1.62	12.13 ± 0.56^B^	13.06 ± 1.71	12.58 ± 1.76	12.31 ± 1.08^D^	11.54 ± 0.29	11.13 ± 1.15^F^	12.25 ± 0.76

## References

[1] Torabinejad M, Corr R, Handysides R, Shabahang S (2009). Outcomes of nonsurgical retreatment and endodontic surgery: a systematic review. *Journal of Endodontics*.

[2] Friedman S, Moshonov J, Trope M (1992). Efficacy of removing glass ionomer cement, zinc oxide eugenol, and epoxy resin sealers from retreated root canals. *Oral Surgery Oral Medicine and Oral Pathology*.

[3] Schirrmeister JF, Wrbas K-T, Meyer KM, Altenburger MJ, Hellwig E (2006). Efficacy of different rotary instruments for gutta-percha removal in root canal retreatment. *Journal of Endodontics*.

[4] Taşdemir T, Yildirim T, Çelik D (2008). Comparative study of removal of current endodontic fillings. *Journal of Endodontics*.

[5] Bodrumlu E, Er O, Kayaoglu G (2008). Solubility of root canal sealers with different organic solvents. *Oral Surgery, Oral Medicine, Oral Pathology, Oral Radiology and Endodontology*.

[6] Nekoofar MH, Ghandi MM, Hayes SJ, Dummer PMH (2006). The fundamental operating principles of electronic root canal length measurement devices. *International Endodontic Journal*.

[7] Uzun O, Topuz O, Tinaz C, Nekoofar MH, Dummer PMH (2008). Accuracy of two root canal length measurement devices integrated into rotary endodontic motors when removing gutta-percha from root-filled teeth. *International Endodontic Journal*.

[8] Erdemir A, Eldeniz AU, Ari H, Belli S, Esener T (2007). The influence of irrigating solutions on the accuracy of the electric apex locator facility in the tri auto ZX handpiece. *International Endodontic Journal*.

[9] Uzun Ö, Topuz Ö, Tinaz AC, Alaçam T (2007). Apical accuracy of two apex-locating handpieces in root canal retreatments of root-end resected teeth. *Journal of Endodontics*.

[10] Topuz Ö, Uzun Ö, Tinaz AC, Bodrumlu E, Görgül G (2008). Accuracy of two apex-locating handpieces in detecting simulated vertical and horizontal root fractures. *Journal of Endodontics*.

[11] Thomas AS, Hartwell GR, Moon PC (2003). The accuracy of the root ZX electronic apex locator using stainless-steel and nickel-titanium files. *Journal of Endodontics*.

[12] Kaufman AY, Keila S, Yoshpe M (2002). Accuracy of a new apex locator: an in vitro study. *International Endodontic Journal*.

[13] American Association of Endodontics http://www.aae.org/glossary/.

[14] Silveira LFM, Petry FV, Martos J, Neto JBC (2011). In vivo comparison of the accuracy of two electronic apex locators. *Australian Endodontic Journal*.

[15] Özsezer E, Inan U, Aydin U (2007). In vivo evaluation of ProPex electronic apex locator. *Journal of Endodontics*.

[16] Ricucci D, Langeland K (1998). Apical limit of root canal instrumentation and obturation, part 2. A histological study. *International Endodontic Journal*.

[17] Jenkins JA, Walker 3rd. WA, Schindler WG, Flores CM (2001). An in vitro evaluation of the accuracy of the root ZX in the presence of various irrigants. *Journal of Endodontics*.

[18] Czerw RJ, Fulkerson MS, Donnelly JC, Walmann JO (1995). In vitro evaluation of the accuracy of several electronic apex locators. *Journal of Endodontics*.

[19] Venturi M, Breschi L (2005). A comparison between two electronic apex locators: an in vivo investigation. *International Endodontic Journal*.

[20] Jung I-Y, Yoon B-H, Lee S-J, Lee SJ (2011). Comparison of the reliability of “0.5” and “aPEX” mark measurements in two frequency-based electronic apex locators. *Journal of Endodontics*.

[21] Gulabivala K, Stock CJR, Gulabivala K, Walker RT, Stock CJR (2004). Root canal system preparation. *Endodontics*.

[22] Takahashi CM, Cunha RS, de Martin AS, Fontana CE, Silveira CFM, da Silveira Bueno CE (2009). In vitro evaluation of the effectiveness of protaper universal rotary retreatment system for gutta-percha removal with or without a solvent. *Journal of Endodontics*.

[23] McDonald MN, Vire DE (1992). Chloroform in the endodontic operatory. *Journal of Endodontics*.

[24] Sae-Lim V, Rajamanickam I, Lim BK, Lee HL (2000). Effectiveness of profile .04 taper rotary instruments in endodontic retreatment. *Journal of Endodontics*.

[25] ElAyouti A, Kimionis I, Chu A-L, Löst C (2005). Determining the apical terminus of root-end resected teeth using three modern apex locators: a comparative ex vivo study. *International Endodontic Journal*.

[26] Ebrahim AK, Yoshioka T, Kobayashi C, Suda H (2006). The effects of file size, sodium hypochlorite and blood on the accuracy of root ZX apex locator in enlarged root canals: an in vitro study. *Australian Dental Journal*.

[27] Ebrahim AK, Wadachi R, Suda H (2007). An in vitro evaluation of the accuracy of dentaport ZX apex locator in enlarged root canals. *Australian Dental Journal*.

[28] Kang J-A, Kim SK (2008). Accuracies of seven different apex locators under various conditions. *Oral Surgery, Oral Medicine, Oral Pathology, Oral Radiology and Endodontology*.

[29] Herrera M, Ábalos C, Lucena C, Jiménez-Planas A, Llamas R (2011). Critical diameter of apical foramen and of file size using the root ZX apex locator: an in vitro study. *Journal of Endodontics*.

[30] Briseño-Marroquín B, Frajlich S, Goldberg F, Willershausen B (2008). Influence of instrument size on the accuracy of different apex locators: an in vitro study. *Journal of Endodontics*.

